# Supplementary figures and images for: A novel method for in silico assessment of Methionine oxidation risk in monoclonal antibodies: Improvement over the 2-shell model
Source: PLoS One. 2022 Dec 29;17(12):e0279689. doi: 10.1371/journal.pone.0279689 (PMC9799309; doi:10.1371/journal.pone.0279689)

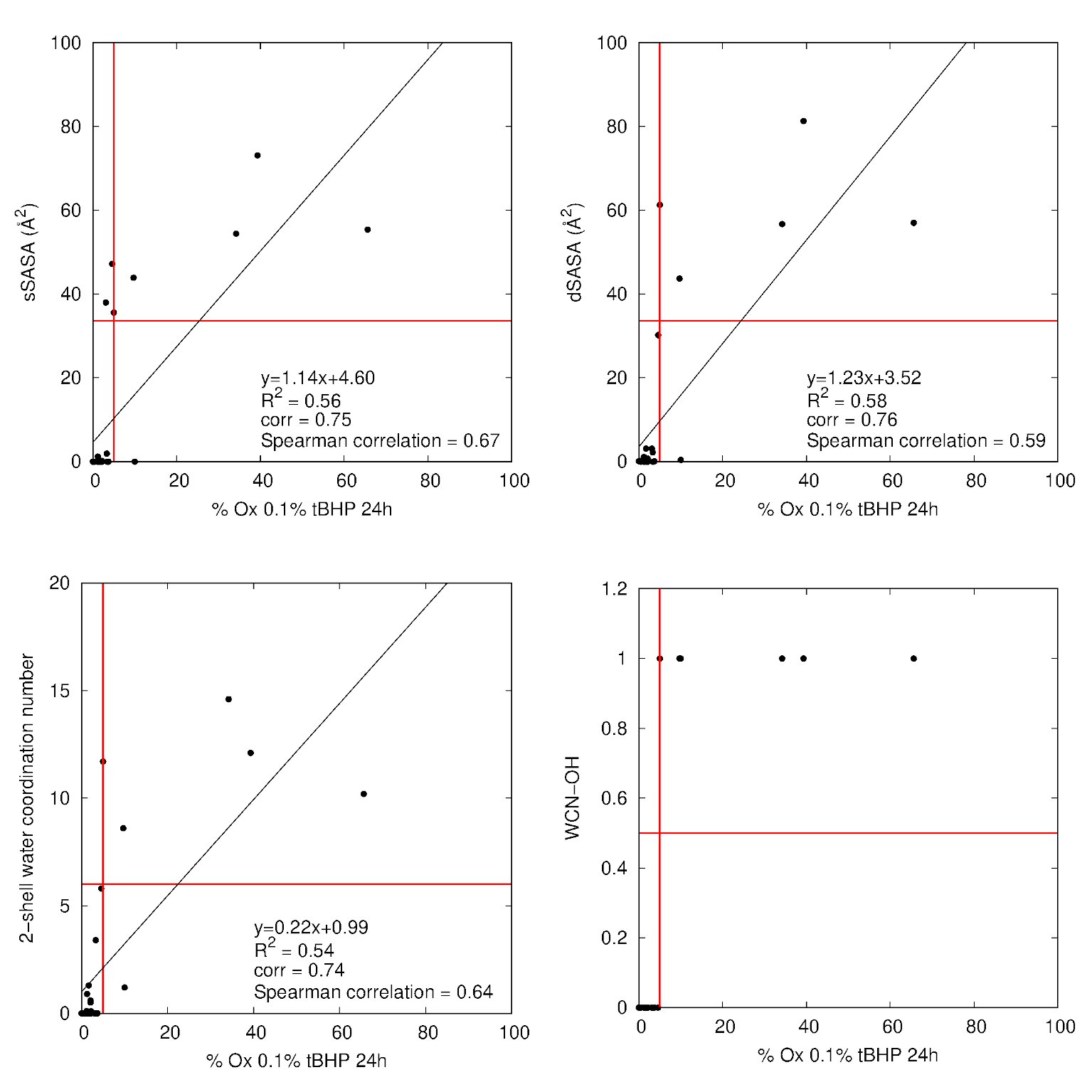

Supplement: S1 Fig — Experimental oxidation level and calculated descriptor for each methionine in consideration are shown in the plot. Vertical red lines indicate oxidation levels measured as ≥ 5%, horizontal red lines indicate threshold values for the different descriptors. (TIF) [file pone.0279689.s001.tif]
