# Supplementary material for: A novel method for in silico assessment of Methionine oxidation risk in monoclonal antibodies: Improvement over the 2-shell model
Source: PLoS One. 2022 Dec 29;17(12):e0279689. doi: 10.1371/journal.pone.0279689 (PMC9799309; doi:10.1371/journal.pone.0279689)
Supplement: S1 Table — Oxidation levels measured as ≥ 5% are shown in red. (DOCX) [file pone.0279689.s002.docx]

|  |  | position | % Ox  0.1% tBHP 24h | Predicted Ox  sSASA | Predicted Ox  dSASA | Predicted Ox  WCN | Predicted Ox  WCN-OH |
| --- | --- | --- | --- | --- | --- | --- | --- |
| mAb2 | Met#4 | HFR2 | 0.4 | 0 | 0 | 0 | 0 |
|  | Met#5 | HFR3 | N.A. | 0 | 0 | 0 | 0 |
| mAb4 | Met#11 | LFR2 | 0.3 | 0 | 0 | 0 | 0 |
|  | Met#12 | HFR2 | N.A. | 0 | 0 | 0 | 0 |
|  | Met#13 | HFR3 | N.A. | 0 | 0 | 0 | 0 |
|  | Met#14 | HFR3 | N.A. | 0 | 0 | 0 | 0 |
|  | Met#15 | CDRH3 | 68.0 | 0 | 0 | 0 | 1 |
| mAb7 | Met#22 | HFR2 | 0.2 | 0 | 0 | 0 | 0 |
|  | Met#23 | HFR3 | N.A. | 0 | 0 | 0 | 0 |
|  | Met#24 | HFR4 | 55.8 | 1 | 1 | 1 | 1 |
| ADC1 | Met#25 | HFR2 | 0.3 | 0 | 0 | 0 | 0 |
| ADC2 | Met#26 | LFR1 | 4.6 | 1 | 0 | 0 | 0 |

**S1 Table. Comparison of experimental results and in silico prediction of oxidation events for 4 proprietary molecules.** Oxidation levels measured as ≥ 5% are shown in red.
