# Supplementary material for: A novel method for in silico assessment of Methionine oxidation risk in monoclonal antibodies: Improvement over the 2-shell model
Source: PLoS One. 2022 Dec 29;17(12):e0279689. doi: 10.1371/journal.pone.0279689 (PMC9799309; doi:10.1371/journal.pone.0279689)
Supplement: S2 Table — (DOCX) [file pone.0279689.s003.docx]

|  |  | sSASA (Å^2^) | dSASA (Å^2^) | WCN | #OH |  |
| --- | --- | --- | --- | --- | --- | --- |
| Abituzumab | H:34 | 3.26 | 1.89 | 1.05 | 0.71 | |
| Abituzumab | H:69 | 0 | 0.01 | 0.04 | 1 | |
| Abituzumab | H:80 | 0 | 0.01 | 0 | 0 | |
| Abituzumab | H:100A | 2.16 | 1.06 | 1.34 | 1 | |
| Dinutuximab | H:19 | 89.94 | 86.37 | 12.03 | 0.75 | |
| Dinutuximab | H:34 | 2.97 | 4.82 | 1.43 | 0 | |
| Dinutuximab | H:80 | 0.38 | 0.08 | 0 | 0.05 | |
| Dinutuximab | H:96 | 49.96 | 15.19 | 5.97 | 2.92 | |
| Duligotuzumab | H:82 | 0 | 0 | 0 | 1 | |
| Duligotuzumab | H:100D | 0.69 | 6.38 | 2.39 | 1 | |
| Eldelumab | H:2 | 76.12 | 54.53 | 11.22 | 0.03 | |
| Eldelumab | H:34 | 0 | 0.24 | 0 | 0 | |
| Eldelumab | H:55 | 117.01 | 115.47 | 15.55 | 0 | |
| Eldelumab | H:82 | 0 | 0.02 | 0 | 0.98 | |
| Eldelumab | H:100G | 0.31 | 2.93 | 3.19 | 1 | |
| Fletikumab | H:48 | 0 | 0.32 | 0.65 | 0.26 | |
| Fletikumab | H:80 | 0 | 0.03 | 0.04 | 0.01 | |
| Fletikumab | H:100J | 0 | 6.42 | 5.32 | 0.91 | |
| Golimumab | H:34 | 0 | 1.54 | 1.92 | 0 | |
| Golimumab | H:51 | 13.6 | 6.97 | 2.84 | 0.1 | |
| Golimumab | H:82 | 0 | 0 | 0 | 0.99 | |
| Golimumab | H:100I | 0 | 0.29 | 0.85 | 1 | |
| Imgatuzumab | H:48 | 0.12 | 0.58 | 0.03 | 0.76 | |
| Imgatuzumab | H:80 | 1.5 | 0.16 | 0 | 0.01 | |
| Imgatuzumab | H:100C | 6.62 | 0.01 | 1.6 | 1 | |
| Lintuzumab | H:34 | 7.43 | 0.34 | 0 | 0.96 | |
| Lintuzumab | H:80 | 0 | 0.01 | 0 | 0 | |
| Lintuzumab | H:99 | 5.94 | 0.04 | 0.39 | 0 | |
| Lirilumab | H:48 | 0 | 0.02 | 0 | 0.91 | |
| Lirilumab | H:80 | 0 | 0.31 | 0 | 0 | |
| Lirilumab | H:100F | 1.9 | 1.13 | 1.66 | 1.69 | |
| Natalizumab | H:48 | 0 | 0.14 | 0.1 | 0.13 | |
| Natalizumab | H:80 | 1.46 | 0.15 | 0.01 | 0.09 | |
| Natalizumab | H:100F | 0 | 0.05 | 0.1 | 0.96 | |
| Ofatumumab | H:34 | 0 | 0.03 | 0 | 0 | |
| Ofatumumab | H:82 | 0 | 0.01 | 0 | 0.98 | |
| Ofatumumab | H:100E | 0 | 0.29 | 0.08 | 1 | |
| Tocilizumab | H:69 | 0 | 4.15 | 2.73 | 0.08 | |
| Tocilizumab | H:100B | 0.15 | 2.52 | 2.46 | 2.78 | |
| Tovetumab | H:34 | 0 | 0.02 | 0 | 0 | |
| Tovetumab | H:82 | 0 | 0.02 | 0 | 0.97 | |
| Tovetumab | H:100C | 0 | 2.47 | 3.67 | 1 | |
| Vesencumab | H:34 | 0.17 | 0.37 | 0 | 0 | |
| Vesencumab | H:82 | 0 | 0 | 0 | 1 | |
| Vesencumab | H:100B | 132.22 | 116.89 | 18.57 | 0.62 | |
| Vesencumab | H:100F | 15.64 | 5.03 | 2.8 | 1.94 | |

**S2 Table. Methionine side chain accessibility parameters calculated from MD trajectories for the CST antibodies.**
