# Supplementary material for: A novel method for in silico assessment of Methionine oxidation risk in monoclonal antibodies: Improvement over the 2-shell model
Source: PLoS One. 2022 Dec 29;17(12):e0279689. doi: 10.1371/journal.pone.0279689 (PMC9799309; doi:10.1371/journal.pone.0279689)
Supplement: S3 Table — (DOCX) [file pone.0279689.s004.docx]

|  |  |  | sSASA (Å^2^) | dSASA (Å^2^) | WCN | #OH |  |
| --- | --- | --- | --- | --- | --- | --- | --- |
| mAb1 | Met#1 | LFR1 | 0 | 3.06 | 1.32 | 0.99 | |
|  | Met#2 | HFR2 | 0 | 0.02 | 0 | 0 | |
|  | Met#3 | HFR3 | 0 | 0.01 | 0 | 0.97 | |
| mAb2 | Met#4 | HFR2 | 0 | 0.06 | 0.04 | 0.51 | |
|  | Met#5 | HFR3 | 0 | 0.12 | 0 | 0.1 | |
| mAb3 | Met#6 | LFR1 | 0 | 0.07 | 0.92 | 1.02 | |
|  | Met#7 | HFR2 | 54.41 | 56.72 | 14.58 | 0.17 | |
|  | Met#8 | HFR2 | 0.19 | 0.7 | 0.56 | 0.28 | |
|  | Met#9 | HFR3 | 43.87 | 43.72 | 8.63 | 1.01 | |
|  | Met#10 | HFR4 | 55.4 | 57.04 | 10.21 | 0.86 | |
| mAb4 | Met#11 | LFR2 | 1.89 | 2.23 | 3.44 | 0.15 | |
|  | Met#12 | HFR2 | 0 | 0.04 | 0.46 | 0.99 | |
|  | Met#13 | HFR3 | 0 | 0 | 0.02 | 0.95 | |
|  | Met#14 | HFR3 | 1.2 | 1.08 | 0 | 0 | |
|  | Met#15 | HCDR3 | 0 | 0.4 | 1.16 | 3.14 | |
| mAb5 | Met#16 | HFR2 | 0 | 0.04 | 0.01 | 0 | |
|  | Met#17 | HFR3 | 0 | 0.01 | 0.06 | 0.84 | |
|  | Met#18 | HCDR3 | 37.96 | 3.13 | 0 | 0 | |
| mAb6 | Met#19 | LFR1 | 0 | 0.12 | 0.09 | 1.21 | |
|  | Met#20 | LFR1 | 35.55 | 61.34 | 11.73 | 0.31 | |
|  | Met#21 | HFR2 | 0 | 0.01 | 0.02 | 1 | |
| mAb7 | Met#22 | HFR2 | 0 | 0 | 0.01 | 0.67 | |
|  | Met#23 | HFR3 | 0 | 0.02 | 0.01 | 0.98 | |
|  | Met#24 | HFR4 | 73.11 | 81.32 | 12.14 | 0.99 | |
| ADC1 | Met#25 | HFR2 | 0 | 0.01 | 0 | 0.43 | |
| ADC2 | Met#26 | LFR1 | 47.18 | 30.16 | 5.84 | 0.31 | |

**S3 Table. Methionine side chain accessibility parameters calculated from MD trajectories for the AbbVie antibodies.**
