# Supplementary material for: A novel method for in silico assessment of Methionine oxidation risk in monoclonal antibodies: Improvement over the 2-shell model
Source: PLoS One. 2022 Dec 29;17(12):e0279689. doi: 10.1371/journal.pone.0279689 (PMC9799309; doi:10.1371/journal.pone.0279689)
Supplement: S1 File — (DOCX) [file pone.0279689.s005.docx]

A novel method for *in silico* assessment of Methionine oxidation risk in monoclonal antibodies: improvement over the 2-shell model.

**Supporting Information**

**Materials and Methods**

**Oxidation stress**

mAb2, mAb4, mAb7, ADC1, ADC2 were rebuffered in 1X PBS buffer pH 7.4 (Gebco) using Zeba spin 0.5 mL desalting columns 40 MWCO (Thermo Fischer (Dreieich, Germany) as described by the manufacturer. Sample concentration was checked using a Nanodrop spectrophotometer (Thermo Scientific, Dreieich, Germany) before further dilution to 2 mg/ml.

To identify oxidation sites, 100 µl of each sample were aliquoted in 1.5 Eppendorf cups and incubated with 0.1% TBHP (Sigma Aldrich, Steinheim, Germany) for 24 hrs at 25°C in the dark using a thermomixer (Eppendorf, Hamburg, Germany).

After incubation time, samples were rebuffered again to PBS to remove the oxidizing agent and frozen for further analysis.

**Digestion**

Samples were digested using a MicroLab STARlet automated liquid handler (Hamilton, Reno, USA). Briefly, each sample was diluted to 1 mg/ml in denaturing buffer (8M Guanidine-HCl, 400 mM Tris, pH 8.0) and reduced in the presence of 20 mM DTT (Sigma Aldrich, Steinheim, Germany) for 1 hr at room temperature. After alkylation in 40 mM Iodoacetic acid (Sigma Aldrich, Steinheim, Germany) for 1 hr in the dark, reaction was quenched in 30 mM DTT and 1 mL PhyTips 10 K cutoff (PhyNexus, San Jose, USA) were used for buffer exchange to hydrolysis buffer (50 mM TRIS pH 7.5). Eluted samples were ultimately digested for 2 hours at room temperature using 1:25 enzyme:protein Trypsin/LysC mix (Promega, Madison, USA). After incubation, samples were acidified using 5% formic acid (Sigma Aldrich, Steinheim, Germany) to quench proteolysis reaction and frozen for further analysis.

**Peptide Mapping**

Peptides generated during proteolysis were separated on a H-Class UPLC (Agilent, Santa Clara, USA) using an ACQUITY BEH 300 C18 column (1.7 µm particle size, 2.1 mm diameter and 150 mm length) (Agilent, Santa Clara, USA) at 65 °C. All solvents used for chromatographic separation were LC/MS grade and purchased from Sigma-Aldrich (Steinheim, Germany). LC/MS water was purchased from Honeywell-Fluka (Seelze, Germany). The solvents used for chromatographic separation were 0.8% (v/v) FA, 0.2% TFA (v/v) in MS grade water (mobile phase A) and 0.8% (v/v) FA, 0.2% TFA in MS grade water in acetonitrile (mobile phase B). Samples were eluted using the following linear gradient: 0.0−1.0 min 1-10% B, 1.0−7.0 min 10−40% B, 7.0-7.9 min 40− 50% B, 7.9-8.0 min 50−90% B, 8.0−8.5 min 90% B, 8.5-9.0 min 90−1% B, 9.0-9.5 min 1%-90% B, 9.5-10 min 90%-1% B, 10.0-10.5 min 1%-90% B, 10.5-11.0 min 90% B, 11.0-11.1 min 90%-1% B, 11.1-15.0 min 1% B, 38.1-50.0 min 1%B with a constant flow rate of 0.450 ml/min.

**Mass spectrometric analysis and oxidation quantification**

Eluted peptides were sprayed by an HESI-source with a spray voltage set at 3.5 kV, capillary temperature 300°C, aux gas heater temperature 430°C, sheath gas and auxiliary gas flow rates of 50 and 15, respectively, in a Q-Exactive Plus mass spectrometer (Thermo Scientific, Dreieich, Germany). Acquisition was performed in a restricted time window between 1-9 min, otherwise the flow was diverted to waste. Full MS scan was set at microscan 1, resolution 70000, ACG target 3e6, maximum IT 50 ms and scan range 200 to 2000 m/z.

dd-MS2 was set at microscan 1, resolution of 17500, AGC target 1e5 and maximum IT 150 ms and an isolation windows of 2.0 m/z in a top-5 method.

Raw files were analysed using Byo from the PMI suite, version 3.6 (Protein Metrics, Cupertino, USA).

The following parameters were used for the peptide identification in Byonics: arginine (R) and lysine (K) were selected as C-terminal cleavage sites and a maximum number of two missed cleavages was allowed. Precursor mass tolerance of 10 ppm and fragment mass tolerance of 30 ppm were used. Carboxymethylation was set as the fixed modification on cysteine and oxidation and dioxidation were set as variable modification for methionine (M). Oxidation quantification was ultimately performed in Byologic. For each sample, sequence coverage was always checked before data analysis.

Peptides were manually verified, and the percentage of oxidation was automatically quantified by the software.
