# Supplementary material for: A novel method for in silico assessment of Methionine oxidation risk in monoclonal antibodies: Improvement over the 2-shell model
Source: PLoS One. 2022 Dec 29;17(12):e0279689. doi: 10.1371/journal.pone.0279689 (PMC9799309; doi:10.1371/journal.pone.0279689)
Supplement: Attachment — Submitted filename: Response to Reviewers.pdf [file pone.0279689.s006.pdf]

Davide Tavella, PhD
100 Research Drive
Worcester, MA 01605
Phone: (508) 410-2932
E-mail: davide.tavella@abbvie.com

November 19, 2022

Dear Dr. Jimenez Del Val,

We were pleased to have an opportunity to revise our manuscript entitled “A novel method for *in silico* assessment of Methionine oxidation risk in monoclonal antibodies: improvement over the 2-shell model”. In the revised manuscript, we have carefully considered the editor’s and reviewer’s suggestions and we adjusted and added some paragraphs accordingly. Moreover, we included more details about the data calculated form the molecular dynamics simulations in the supplementary information. In the rest of the letter, we address and respond to each point raised by the reviewer. The responses to the reviewer's comments are below and are color-coded as follows: a) Comments from editors or reviewers are colored in red b) Our responses are shown under each comment as normal text. The reviewer’s comments were very helpful overall, and we are appreciative of such constructive feedback on our original submission. After addressing the issues raised, we feel the quality of the paper is much improved.

Best regards,

Davide Tavella
Senior Scientist I
Biologics Discovery Sciences

abbvie

Responses to editor's comments:

1. Please ensure that your manuscript meets PLOS ONE's style requirements, including those for file naming. The PLOS ONE style templates can be found at https://journals.plos.org/plosone/s/file?id=wjVg/PLOSOne_formatting_sample_main_body.pdf and https://journals.plos.org/plosone/s/file?id=ba62/PLOSOne_formatting_sample_title_authors_affiliations.pdf

We have corrected the format of the authors' affiliation as per template instructions. We have changed the style and font size of sections' and subsections' titles. We have moved the captions of all figures at the end of the sections where they have been firstly referred to, and we have adjusted the captions' style. We have moved all tables at the end of the sections where they have been firstly referred to. We have attached to this submission all the figures as separated files named accordingly to the style templates. Figures have been assessed by PACE. We hope that it now fits the style requirements, as described in the referred templates.

2. We note that the grant information you provided in the 'Funding Information' and 'Financial Disclosure' sections do not match.
When you resubmit, please ensure that you provide the correct grant numbers for the awards you received for your study in the 'Funding Information' section.

We have changed the "Disclosure" section in order to include the sources of funding for the work included in this submission. No grants were awarded to any authors. All authors participated to this work as employees of AbbVie or Schrödinger, and AbbVie sponsored and funded the study.

3. Thank you for stating the following financial disclosure:
"The design, study conduct, and financial support for this research were provided by AbbVie. AbbVie participated in the interpretation of data, review, and approval of the publication. Davide Tavella, Christopher Negron, David R. Ouellette, Raffaella Garofalo and Jianwen Xu are employees of AbbVie and own AbbVie stock. Peter M. Ihnat was an employee of AbbVie at the time of this study. . Desmond, Prime, Maestro and BioLuminate are products sold by Schrodinger, Inc. Eliud O. Oloo and Kai Zhu performed this research as employees of Schrödinger, Inc."
Please state what role the funders took in the study. If the funders had no role, please state: "The funders had no role in study design, data collection and analysis, decision to publish, or preparation of the manuscript."
If this statement is not correct you must amend it as needed.
Please include this amended Role of Funder statement in your cover letter; we will change the online submission form on your behalf.

We have changed the "Disclosure" section as follow:
AbbVie sponsored and funded the study, contributed to the design, participated in the collection, analysis, and interpretation of data, and in writing, reviewing, and approval of the final publication. Davide Tavella, Christopher Negron, David R. Ouellette, Raffaella Garofalo

and Jianwen Xu are employees of AbbVie and may own AbbVie stock. Peter M. Ihnat was an employee of AbbVie at the time of this study. Desmond, Prime, Maestro and BioLuminate are products sold by Schrödinger, Inc. Schrödinger, Inc. provided support in the form of salary for authors Eliud O. Oloo and Kai Zhu but did not have any additional role in the study design, data collection and analysis, decision to publish or preparation of the manuscript. The specific roles of these authors are articulated in the "author contributions".
The roles of the two funders, AbbVie and Schrödinger, have been explicitly stated as per provided instructions.

4. In your Data Availability statement, you have not specified where the minimal data set underlying the results described in your manuscript can be found. PLOS defines a study's minimal data set as the underlying data used to reach the conclusions drawn in the manuscript and any additional data required to replicate the reported study findings in their entirety. All PLOS journals require that the minimal data set be made fully available. For more information about our data policy, please see http://journals.plos.org/plosone/s/data-availability.
Upon re-submitting your revised manuscript, please upload your study's minimal underlying data set as either Supporting Information files or to a stable, public repository and include the relevant URLs, DOIs, or accession numbers within your revised cover letter. For a list of acceptable repositories, please see http://journals.plos.org/plosone/s/data-availability#loc-recommended-repositories. Any potentially identifying patient information must be fully anonymized.
Important: If there are ethical or legal restrictions to sharing your data publicly, please explain these restrictions in detail. Please see our guidelines for more information on what we consider unacceptable restrictions to publicly sharing data: http://journals.plos.org/plosone/s/data-availability#loc-unacceptable-data-access-restrictions. Note that it is not acceptable for the authors to be the sole named individuals responsible for ensuring data access.
We will update your Data Availability statement to reflect the information you provide in your cover letter.

In this work, we have used two experimental datasets to validate the *in silico* predictions of methionine oxidation propensities and to reach the conclusions drawn in the manuscript.
The first dataset, referred to in the manuscript as "clinical stage therapeutic (CST) antibodies dataset", was derived from existing data, which are openly available at https://doi.org/10.1080/19420862.2017.1290753. This is the largest dataset used in this work, counting 14 antibodies and a total of 46 methionines.
The second dataset, referred to in the manuscript as "internal dataset", is derived from antibodies and ADCs currently in development at AbbVie. Therefore, due to its proprietary nature, supporting data cannot be made openly available. This is the smallest dataset used in this work, counting 7 antibodies and 2 ADCs for a total of 26 methionines.
We want to highlight that the conclusions drawn from both datasets are in total agreement and the "internal dataset" serves as a further validation of the methods described in the manuscript. Moreover, in the supporting information S2 Table and S3 Table, we have disclosed more details regarding the calculations derived from the molecular dynamics simulations for both datasets.

abbvie

5. Please include captions for your Supporting Information files at the end of your manuscript, and update any in-text citations to match accordingly. Please see our Supporting Information guidelines for more information: http://journals.plos.org/plosone/s/supporting-information.

We have added captions for Supporting Information Tables and Figures at the end of the manuscript.

6. Please review your reference list to ensure that it is complete and correct. If you have cited papers that have been retracted, please include the rationale for doing so in the manuscript text, or remove these references and replace them with relevant current references. Any changes to the reference list should be mentioned in the rebuttal letter that accompanies your revised manuscript. If you need to cite a retracted article, indicate the article's retracted status in the References list and also include a citation and full reference for the retraction notice.

We have revised the reference list to match the style and format of the journal. We have included a new reference (reference 49 in the final version). No retracted articles are referenced in the manuscript.

Responses to reviewer #2:
Reviewer #2: The article on Met oxidation of MAbs is lucid and interesting. I endorse its ultimate publication as a good contribution to our understanding adventitious met oxidation, both in therapeutic molecules and in the cell.
While I understand the focus on go/no go criteria to drive pharma decisions, well supported with the confusion matrices, I'd prefer some explicit statistical predictions of "oxidation riak" shall we say. Reporting a binary cutoff at a 15%? threshold is nice but why is that the right number? Cna the authors share mnre results from their calculations or present them in an alternate way, even as supporting material?

We agree with the reviewer's points, and thus we included a statistical analysis of the linear correlation between experimentally measured oxidation levels of methionines and parameters calculated from the molecular dynamics simulations, specifically water coordination number and SASA. These results are reported in the modified S1 Fig and discussed in the result section at lines 443-450. The statistical analysis includes R-squared, correlation coefficient and Spearman correlation coefficient.
The choice of 15% threshold is now discussed in the result section at line 281-282.
We added 2 tables, S2 Table and S3 Table in supporting information, with details of the calculations from the molecular dynamics simulations.

A second point concerns MS data, will the new generated be made available in PRIDE or other database? This is important to the study's impact.

As discussed in the response to Editor's comment #4, the dataset indicated as "internal dataset" is derived from antibodies and ADCs currently in development at AbbVie. Therefore, due to its proprietary nature, supporting data cannot be made openly available.
The "clinical stage therapeutic (CST) antibodies dataset" was derived from existing data, which are openly available at https://doi.org/10.1080/19420862.2017.1290753.

abbvie

Lastly I'm wondering about timescales. For calculating time averaged surface excursions of partially buried Met, and thinking about chemical reaction dynamics, what % of Met are available for chemical reaction at any time? Does this fit with the concentrations and reactivity of the oxidation agents used?

We have added a paragraph that address the different time scales involved in the molecular dynamics simulations and the kinetics of the oxidation reaction in the Discussion section at lines 514-525.

Thank you again for your time and effort.

Best regards,

Davide Tavella
Senior Scientist I
Biologics Discovery Science
